# Radial Club Hand Treated by Paley Ulnarization Generation 3: Is This the New Centralization?

**DOI:** 10.3390/children8070562

**Published:** 2021-06-29

**Authors:** Jaroslaw M. Deszczynski, Tomasz Albrewczynski, Claire Shannon, Dror Paley

**Affiliations:** 1 Department of Orthopedics and Rehabilitation, Warsaw Medical University, 02-091 Warsaw, Poland; jdeszczynski@wum.edu.pl; 2 Paley European Institute, 02-972 Warsaw, Poland; talbrewczynski@paleyeurope.com; 3 Paley Orthopedic and Spine Institute, 901 45th St. Kimmel Building, West Palm Beach, FL 33407, USA; cshannon@paleyinstitute.org

**Keywords:** radial club hand, ulnarization, radialization, centralization, radial aplasia, radial longitudinal defect

## Abstract

(1) Background: Patients treated with the two previous generations of ulnarization developed a bump related to the ulnar head becoming prominent on the radial side of the hand. To finally remedy this problem, a third generation of ulnarization was developed to keep the ulnar head contained. While still ulnar to the wrist center, the center of the wrist remains ulnar to the ulnar head, with the ulnar head articulating directly with the trapezoid and when present the trapezium. (2) Methods: Between 2019 and 2021, 22 radial club hands in 17 patients were surgically corrected with this modified version of ulnarization. (3) Results: In all 17 patients, the mean HFA (hand–forearm-angle) correction was 68.5° (range 12.2°–88.7°). The mean ulna growth was 1.3 cm per year (range 0.2–2 cm). There were no recurrent radial deviation deformities more than 15° of the HFA. (4) Conclusions: This new version of ulnarization may solve the problem of the ulna growing past the carpus creating a prominent ulnar bump. The results presented are preliminary but promising. Longer-term follow-up is needed to fully evaluate this procedure.

## 1. Introduction

Radial Club Hand (RCH) is a rare condition occurring in 1:30,000 to 1:100,000 live births [1,2,3]. It is often associated with radial ray deficiency [4,5,6,7]. RCH can be associated with other congenital deformities or can occur as an isolated defect. Children with RCH have functional deficits due to the wrist deviation, lower grip strength, shortness of the forearm and thumb absence or dysfunction [8]. The growth rate in RCH has been shown to be three quarters to half of that of the normal ulna [9]. Centralization of the wrist is the most common surgical approach for the management of RCH [10], despite documentation showing high recurrence of radial deviation, growth arrest of the distal ulnar physis and wrist stiffness [11,12].

Buck-Gramcko [13] described ‘radialization’ in 1985, translocating the carpus to the ulnar side of the ulnar head. The name radialization referred to the ulna becoming a radius. Distal ulnar physeal arrest occurred in 11% and recurrent radial deviation in 7.5%. These are lower than those reported for centralization [13,14].

The senior author (D.P.) described a new procedure called ‘ulnarization’, which is performed through a volar approach with tendon transfer of the flexor carpi ulnaris (FCU) to the dorsum of the wrist [15,16], instead of a dorsal approach with tendon transfer of the flexor carpi radialis (FCR) to the dorsum of the wrist. Ulnarization refers to the direction of movement of the carpus relative to the ulna. Ulnarization is more extensile and permits decompression of the ulnar and median nerves and radial and ulnar arteries. The caput ulnar artery, which is a branch of the ulnar artery, can be visualized and protected, preventing damage to the circulation of the distal ulnar physis and epiphysis [17].

Paley reported 0% recurrent wrist deformity or growth arrest after ulnarization [17]. In longer term follow-up, 10–15% of patients developed gravity-related dynamic ulnar deviation. Furthermore, 80% of all patients developed a bump related to the ulnar head becoming prominent on the radial side. This was not painful but signified that the ulna was growing past the wrist in most cases. For reference, the original ulnarization procedure [15,16] with the use of external fixation will be referred to as ulnarization G1 (Generation 1). To improve upon this, Paley modified the procedure by shortening the ulna [17]. The shortening did not eliminate the ulnar bump in every case and some cases continued to grow past the carpus as in G1. Once again there were no recurrent deformities or growth arrests. For reference, this shall be referred to as ulnarization G2 (Generation 2) [18]. In 2019, Paley modified the procedure again, by releasing the scapholunate ligaments and hinging the scaphoid bone on its distal attachments to move it out of and make room for the ulnar head so that the ulnocarpal joint would be more congruent and stable to axial growth. The preservation of the caput ulna vessels remains as a central tenet of the procedure; the more central location puts the wrist at theoretically greater risk of recurrence as in centralization. The purpose of this study is to report the preliminary results of this third generation of the ulnarization procedure (ulnarization G3) and to describe the surgical procedure in detail so that other surgeons can reliably perform it following the detailed description and illustrations.

## 2. Materials and Methods

Between 2019 and 2021, 22 radial club hands in 17 patients were surgically corrected by the two American co-authors (D.P. and C.S.) using the ulnarization G3 procedure. All surgeries were performed at the Medicover hospital in Warsaw, Poland, and all postoperative care was performed by the two Polish co-authors (J.M.D. and T.A.) (Table 1). The indications for surgery were patients with partial or near complete absence of the radius, with radial deviation of the hand in the RCH position, over the age of 12 months, and with mobile elbows. If the elbow had an extension contracture that could be released and elbow motion restored, it was not considered a contraindication to reconstruction with ulnarization. If the elbow was ankylosed, it was considered a contraindication to surgery. Patients with TAR (thrombocytopenia–absent radius) were all evaluated and managed by a pediatric hematologist. If the preoperative platelet level was less than 100,000, they were given platelet transfusions the morning of surgery and the platelet level repeated. Once it was over 100,000 the surgery could proceed.

There were 12 right and 10 left hands. Twelve were unilateral and 5 bilateral. The mean age at surgery was 24.7 months (range 17–52 months). All postoperative complications from surgery were recorded. Radiographic measurements were made of the hand–forearm angle (HFA), ulnar bow angle and the ulnar length before and after surgery. The HFA was measured between the axis of the long finger metacarpal and a perpendicular line dropped from the midpoint of the distal ulnar physis [19]. The ulnar bow was calculated as the angle formed between the distal articular angle and proximal articular angle. Ulnar length was measured in a straight line between the proximal apophysis to the distal growth plate. Fourteen hands in 10 patients had a follow-up of greater than 12 months since surgery, with the mean being 16.3 months (range 12–19 months). This group had radiographic and clinical assessment for recurrent deformity of the ulna and hand using the same measurements with addition of the combined flexion and extension of the wrist. The latest follow-up measurement of the HFA and ulnar bow as compared to the postoperative measurement was used to assess for recurrent deformity. Length of the ulna in this group was used to assess the growth of the ulna.

### Paley Ulnarization G3 Surgical Technique:

**Figure 1:**
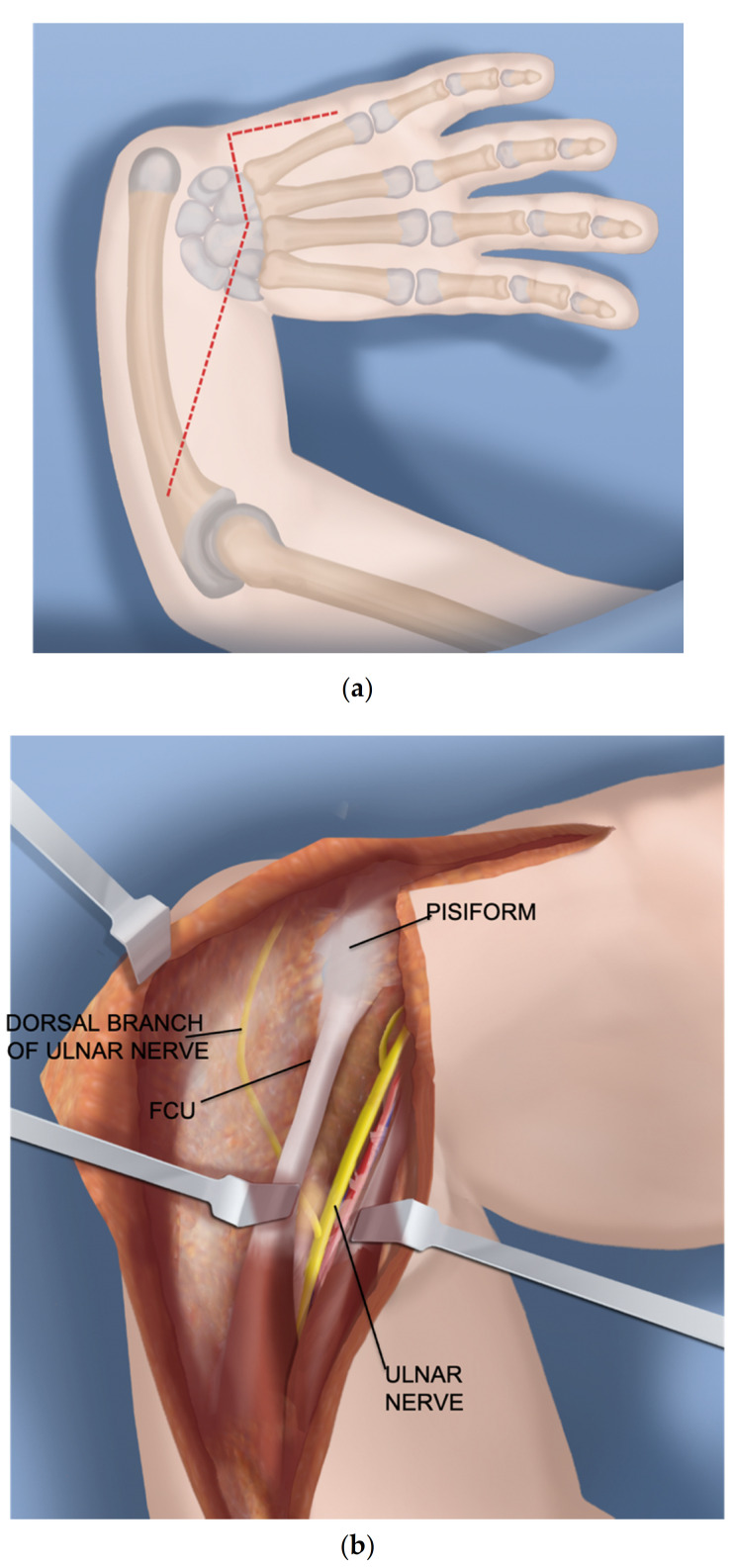

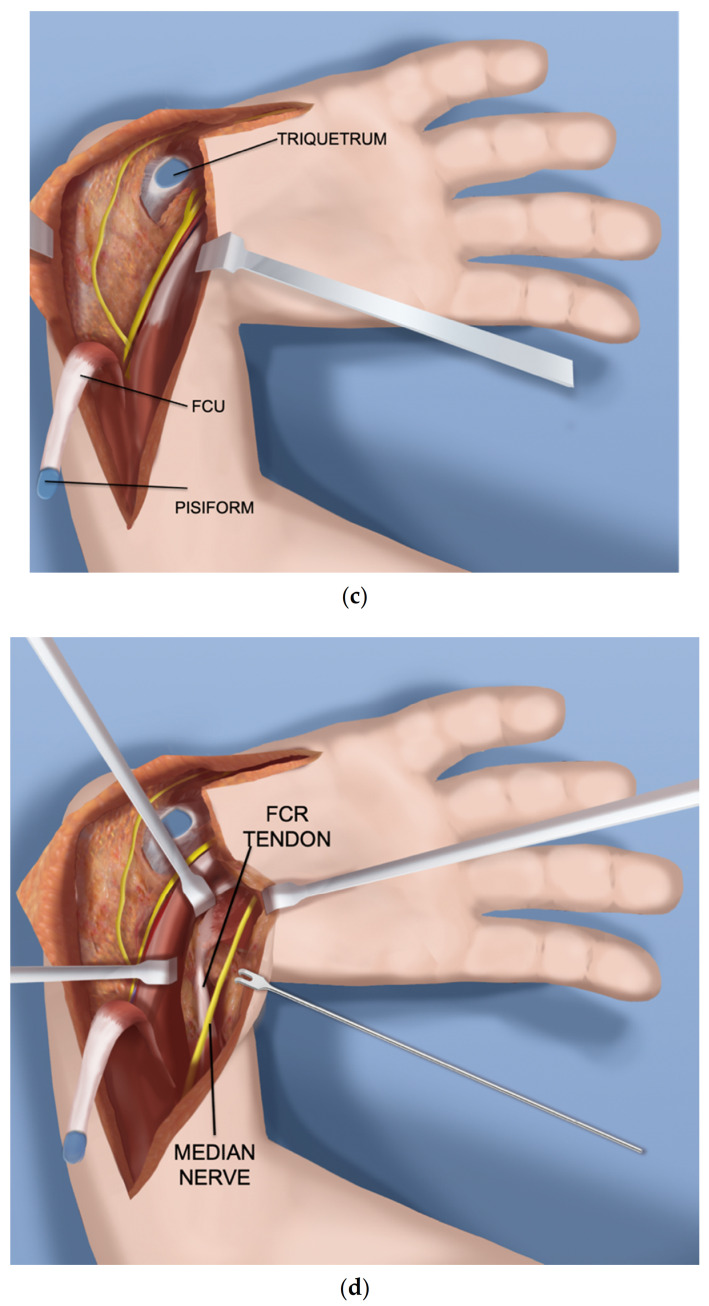

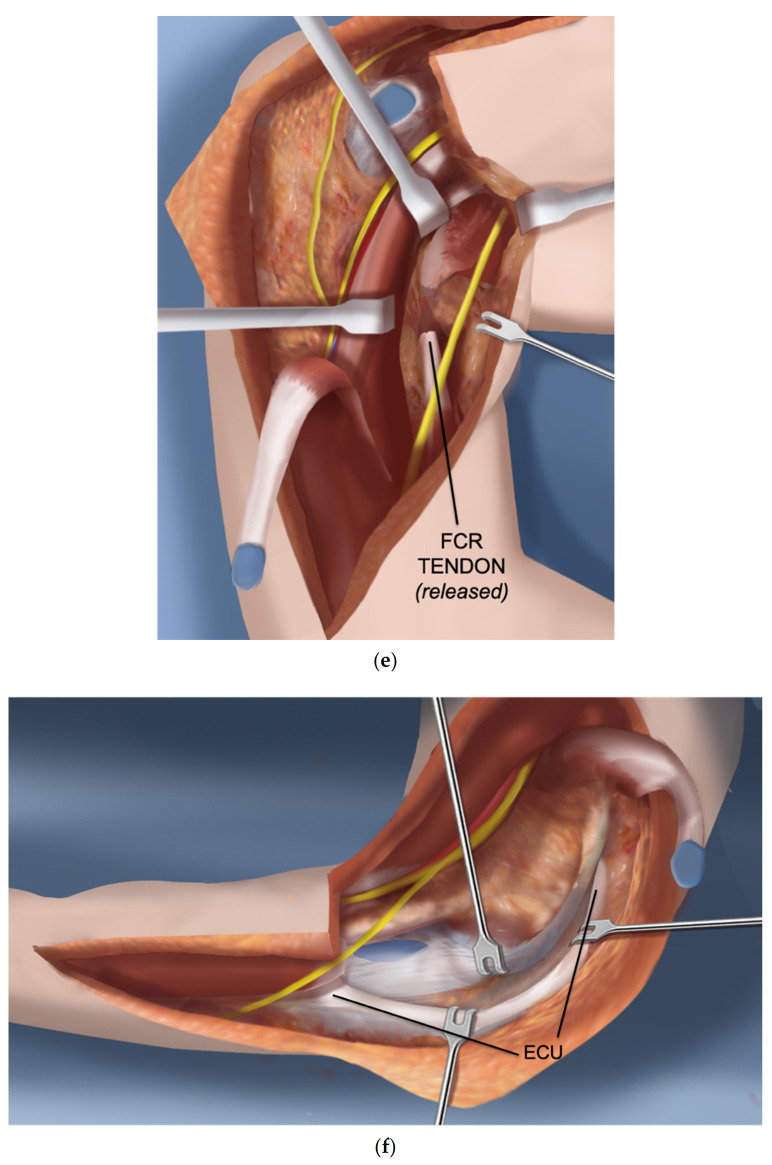

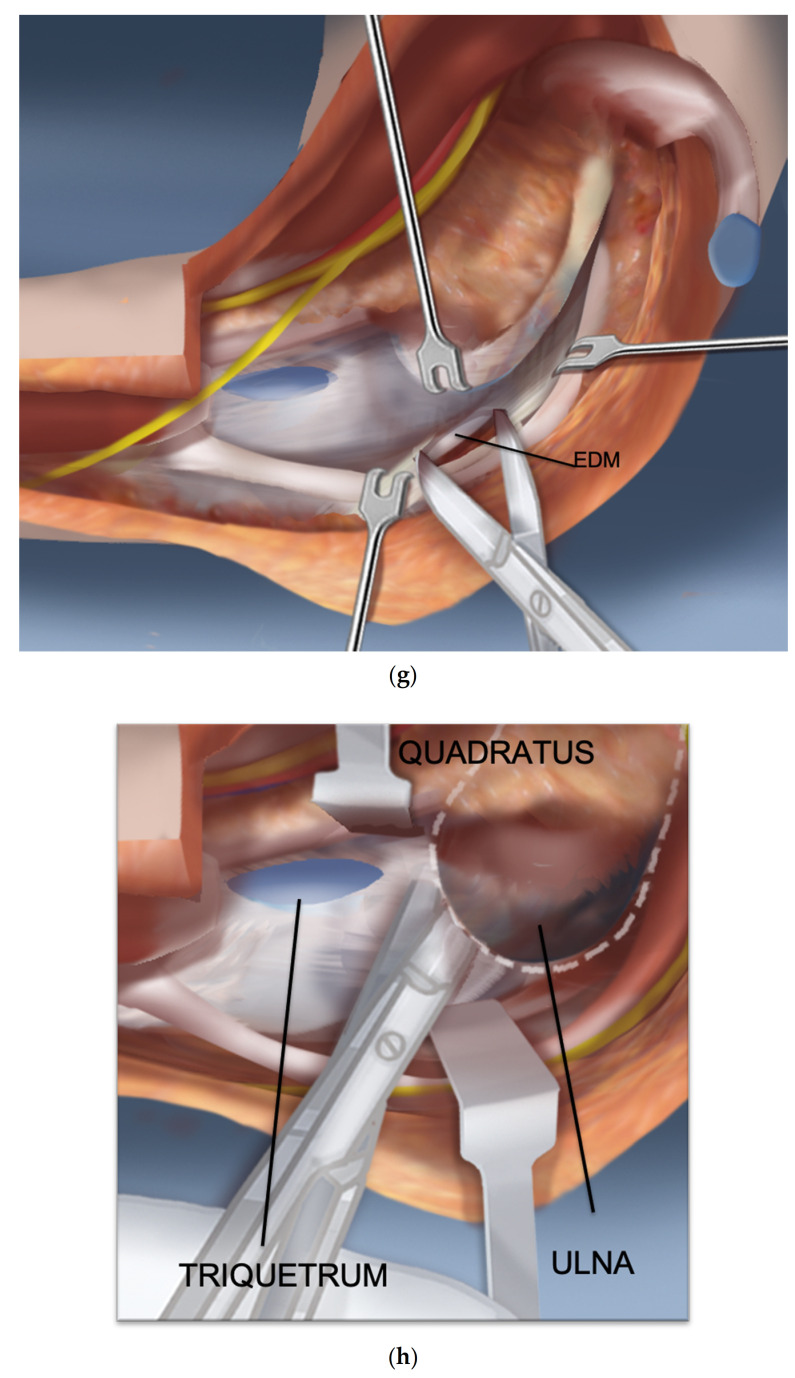

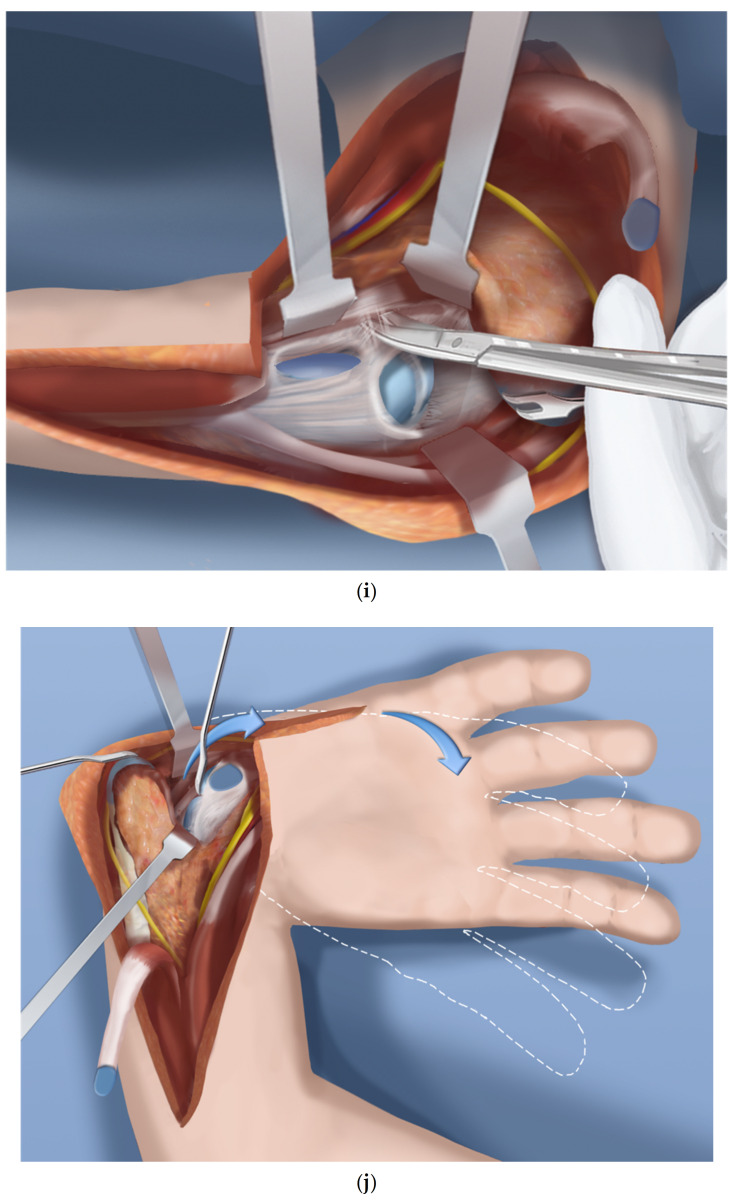

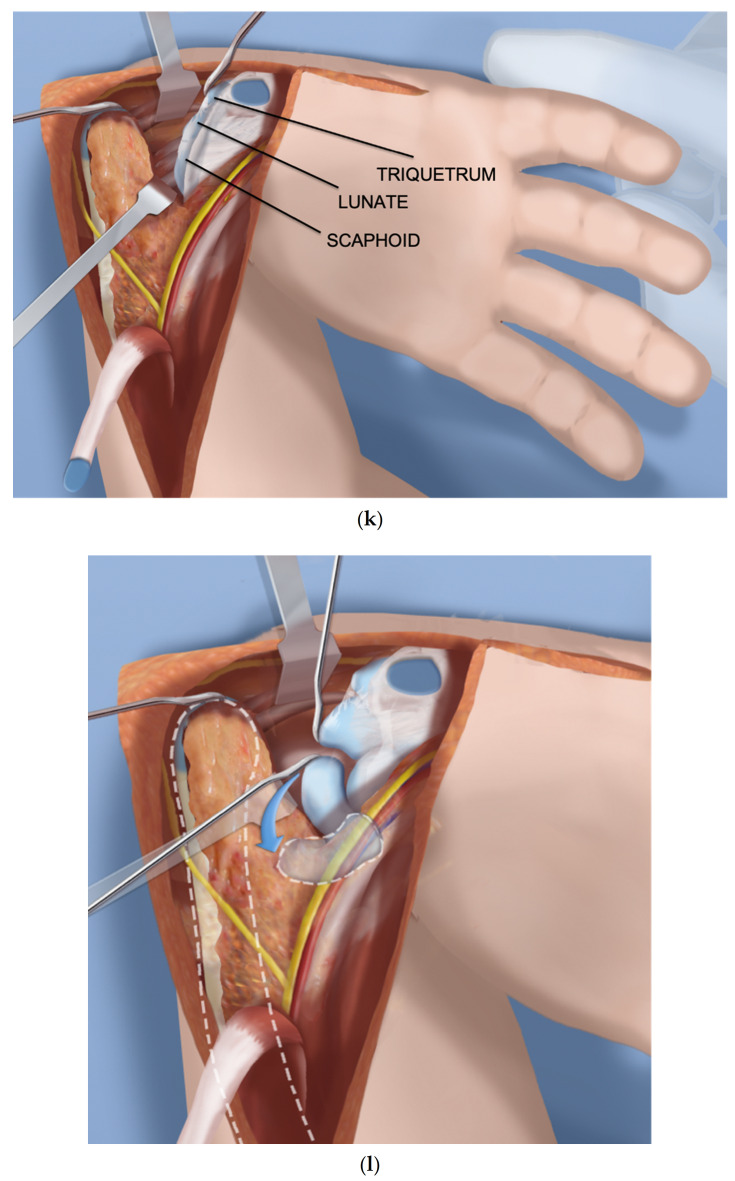

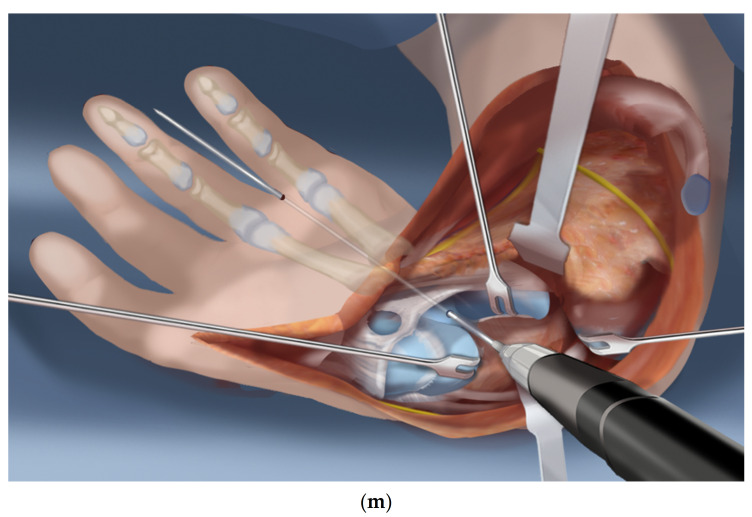
(**a**–**m**) Steps 1–13.

**Figure 2:**
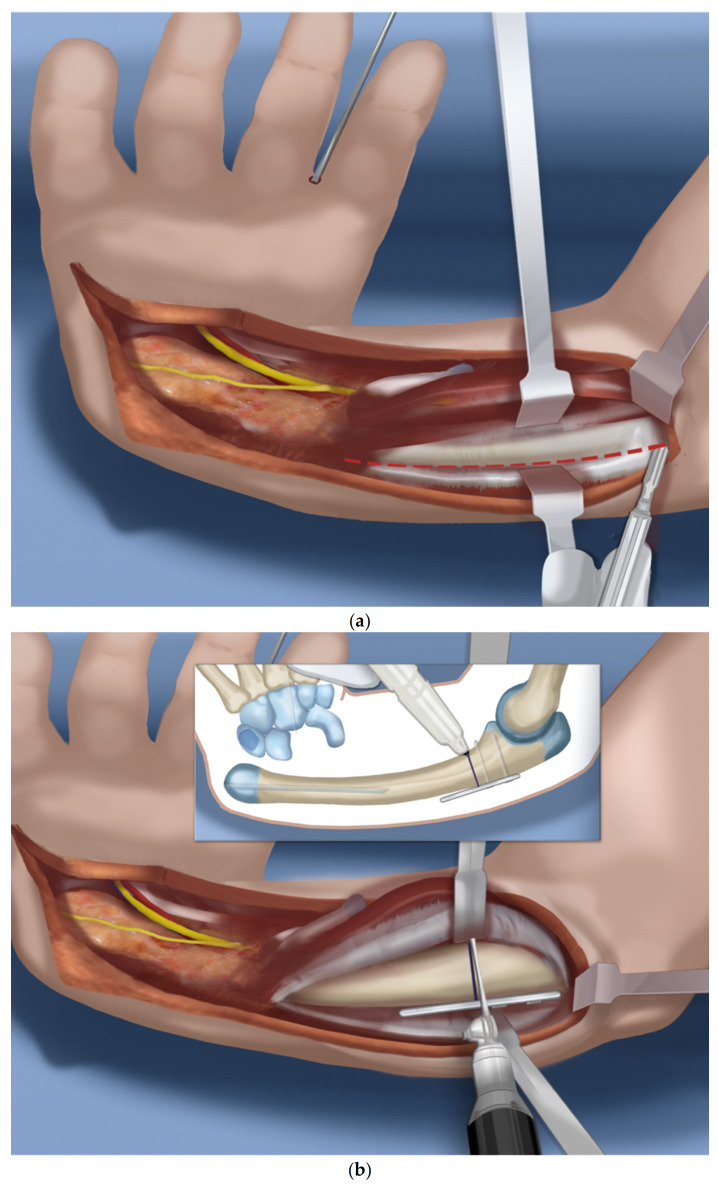

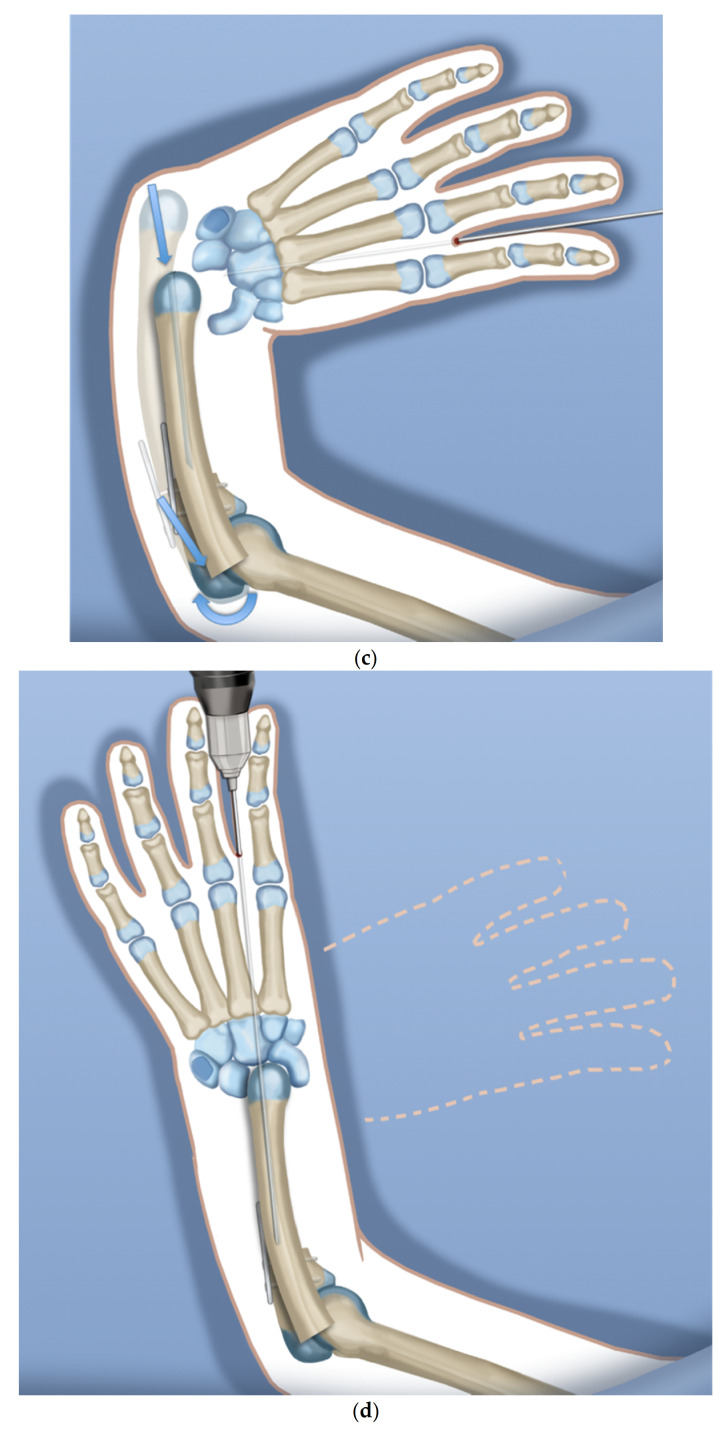

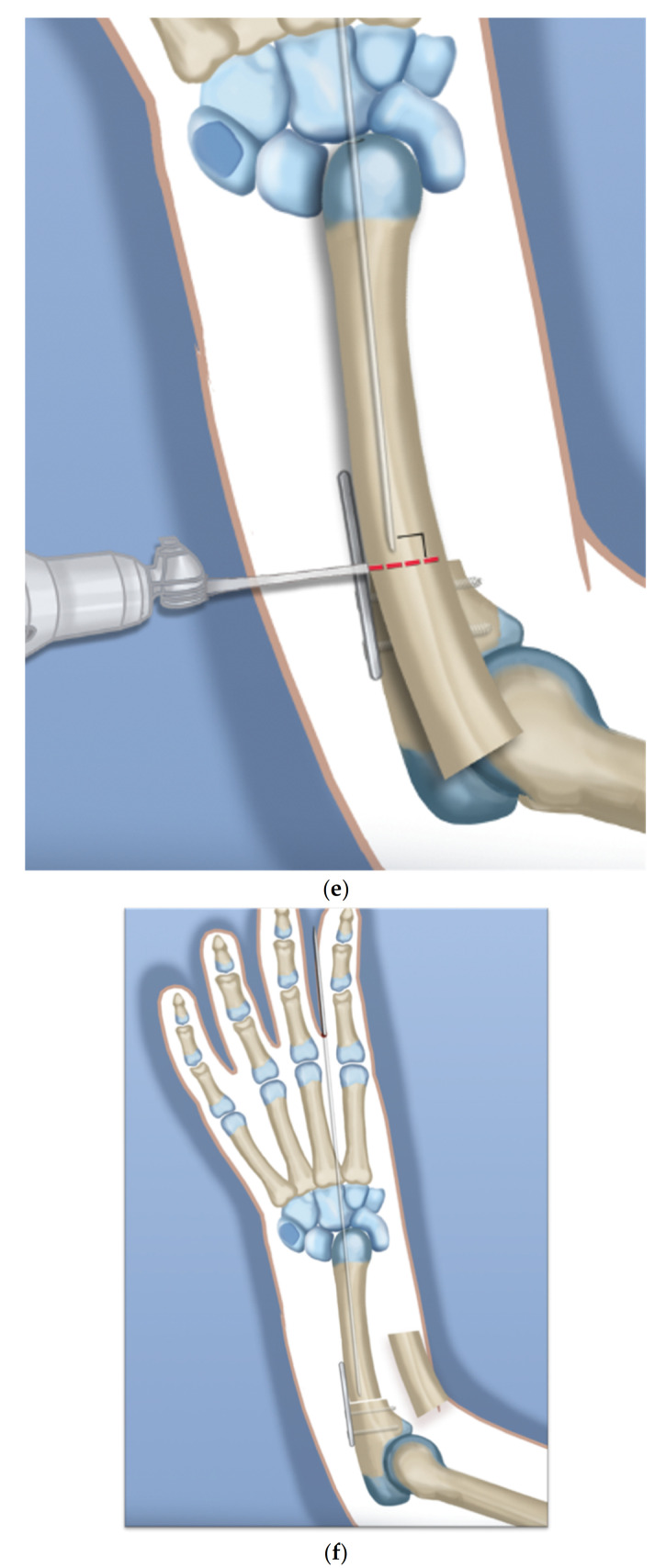

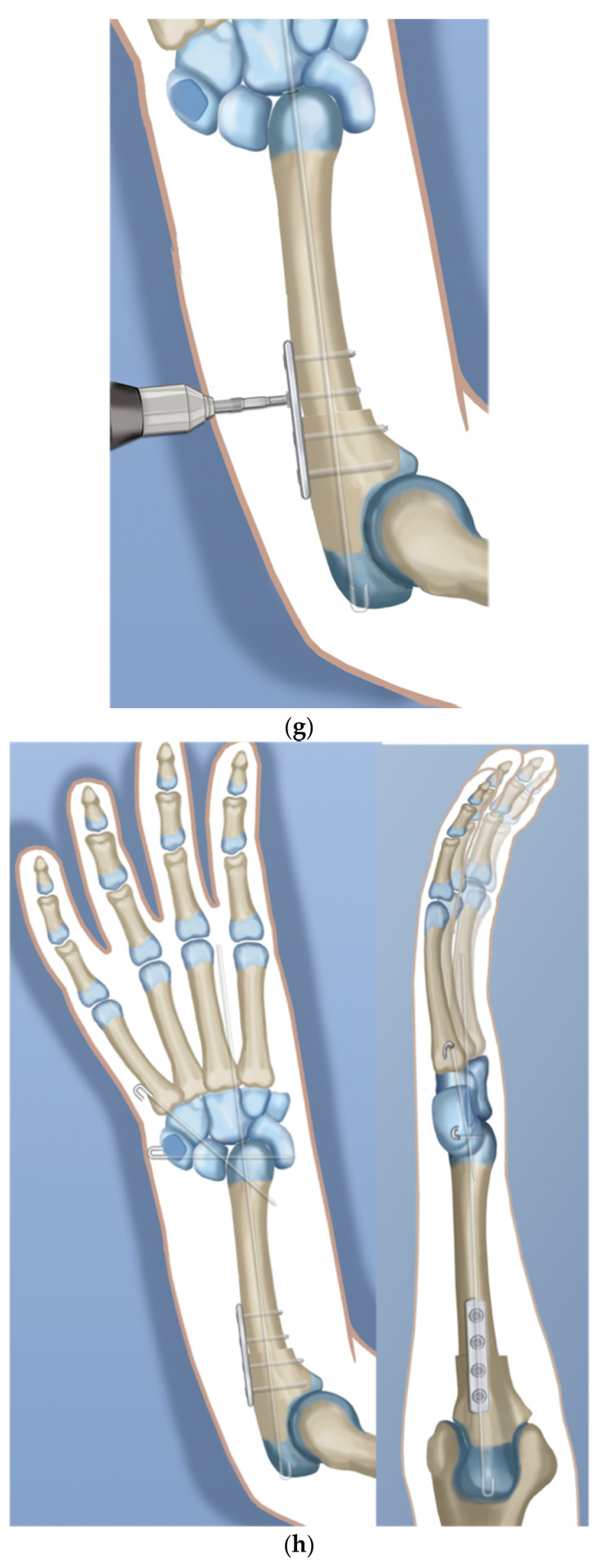
(**a**–**h**) Steps 14–21.

**Figure 3:**
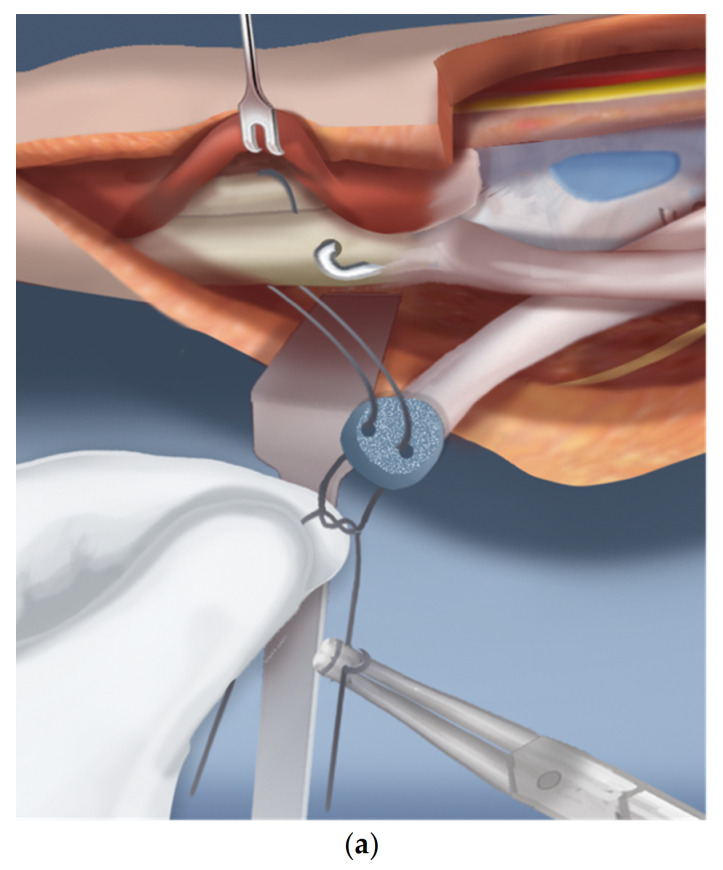

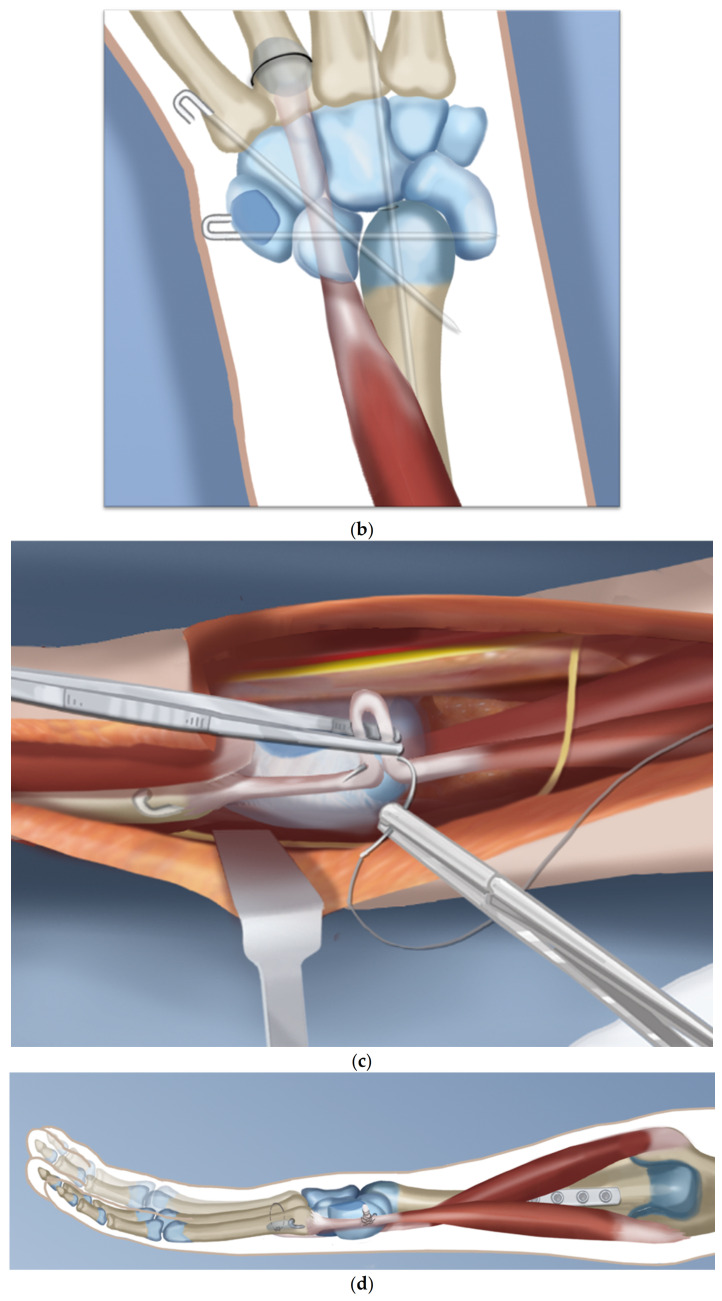
(**a**–**d**) Steps 22–25.

Step 1. After a tourniquet is applied to the arm, a volar Z-shaped incision is made. The middle line of the Z is along the wrist flexor crease. The proximal longitudinal incision runs from the midpoint of the wrist crease towards the ulnar border of the forearm. The distal limb is along the ulnar border of the hand (Figure 1a).
Step 2. A volar fasciotomy is performed. The ulnar neuro-vascular bundle is exposed together with the flexor carpi ulnaris (FCU) tendon. Care is taken not to injure the dorsal branch of the ulnar nerve which passes underneath the FCU (Figure 1b).
Step 3. Decompress the ulnar nerve and the dorsal branch of the ulnar nerve. Detach the pisiform bone from the triquetrum and reflect back the FCU with its muscle (Figure 1c).
Step 4. Expose and decompress the median nerve into the carpal tunnel. Look for an FCR tendon (Figure 1d).
Step 5. Detach the FCR from the carpus. In a TAR syndrome with weak thumb extension, transfer it to the EPL (extensor pollicis longus) or EPB (extensor pollicis brevis) (Figure 1e).
Step 6. On the ulnar side dissect the ECU (extensor carpi ulnaris) free of the ulna to its insertion (Figure 1f).
Step 7. Find the EDM (extensor digiti minimi) on the dorsum of the ulna and free it up proximally and distally (Figure 1g).
Step 8. The ulno-carpal capsule is cut laterally. The dissecting scissors are oriented from the distal to proximal end along the shaft of the ulna. The natural tendency is to dissect from the ulnar to the radial cutting across the lunate. The exposed triquetral side of the piso-triquetral joint helps to orient the anatomy (Figure 1h).
Step 9. It is important to preserve the volar soft tissues anterior and radial to the head of the ulna. A retractor is placed there to protect these tissues that contain the caput ulnar vessels. The capsulotomy is continued in a radial direction on the carpal side (Figure 1i).
Step 10. The volar soft tissue pedicle must be protected at all times during the ulno-carpal capsulotomy (Figure 1j).
Step 11. The carpal bones are released from their capsular connections while preserving the volar flap (Figure 1k).
Step 12. Once the scaphoid is reached, the scapholunate ligaments are released from and the scaphoid is flapped open (Figure 1l).
Step 13. Insert a 1.5 mm wire through the distal row of the carpus after flapping back the scaphoid. This wire exits between the index and middle fingers (Figure 1m).
Step 14. Expose the proximal shaft of the ulna subperiosteally (Figure 2a).
Step 15. Apply a 4-hole locking plate by affixing it with two locking screws to the ulna just proximal to the planned level of the osteotomy and mark the osteotomy level distal to the screws. Predrill the end of the ulna with a 1.8 mm wire to make it easier to pin the hand to the ulna in Step 17 (Figure 2b).
Step 16. After the osteotomy, shorten the ulna and overlap the bone ends. This brings the head of the ulna to the level of the diastasis created between the lunate and the scaphoid (Figure 2c).
Step 17. Advance the wire in the hand into the hole created in the head of the ulna and part way down the shaft of the ulna (Figure 2d).
Step 18. Mark the level of overlap and perform an osteotomy for shortening of the ulna (Figure 2e,f).
Step 19. Advance the ulnar wire across the osteotomy and out the olecranon. Adjust it at the wrist and then cut and bend this wire and bury under the skin. Complete the plate fixation by insertion of the two distal locking screws (Figure 2g).
Step 20. Pin the carpus to the head of the ulna with the plane of the hand in mid pronation-supination using two additional 1.5 mm wires, one transverse and one oblique. Cut and curl the near end of the wires and bury under the skin (Figure 2h).
Step 21. Use a 15 blade to remove the articular surface of the pisiform. Pass the FCU tendon deep to the ECU and EDM and the dorsal cutaneous branch of the ulnar nerve. Pass a 2-0 non-absorbable suture from dorsal to volar through the pisiform. Pass the needle through the interspace between the 3rd and 4th metacarpal bases from dorsal to volar. Then, re-pass the same needle from volar to dorsal between the 4th to 5th metacarpal bases. Pass the needle through the pisiform from volar to dorsal. Secure the pisiform down to the bone by tying the suture (Figure 3a,b).
Step 22. Imbricate the ECU tendon with non-absorbable sutures (Figure 3c,d).
Step 23. Extend the volar fasciotomy of the forearm proximally.
Step 24. The tourniquet is let down. After achieving hemostasis, close the wound over a drain. Excess skin folds on the ulnar side should be resected.
Step 25. A custom molded splint either from cast material or orthoplasty is made in the operating room. The above elbow splint should leave the fingers free to move.

Postoperative management:

The arm is strictly elevated, and circulatory checks and splitting of any circumferential dressing are carried out. Pain control is accomplished with intravenously administered narcotics and then switched to oral medication usually 24 h after surgery and the patient is discharged from hospital. Physical therapy begins on postoperative day one and consists of elbow and finger range of motion (ROM) exercises. After six weeks, replace the long arm splint with a below elbow ulnar gutter splint. The temporary arthrodesis wires of the wrist should be removed after three months. This can be combined with pollicization of the index finger surgery when indicated. Physical therapy for active and passive range of motion of the wrist can begin at this time.

## 3. Results

There were five postoperative wound complications: four superficial and one deep. The four superficial (Cases 1, 3, 5 and 6) were all treated with wet-to-dry dressings and topical antiseptics. In the first six cases, we did not resect redundant skin and allowed it to bunch up on the ulnar side. After experiencing 4/6 wound complications in this group, we began resecting the redundant skin. We did not observe any further superficial wound complications but one patient with bilateral ulnarization had a deep wound infection on the right side (Case 12), treated with intravenous antibiotics and later removal of all hardware, including the plate and wires at 12 weeks, when the bone was healed. This case showed no ulnar growth at one-year follow-up.

There were several electives associated or staged planned procedures performed on this group of patients. All had wires removed from the wrist 12 weeks after the ulnarization. Five had elective pollicization done in conjunction with wire removal or at a separate time. Two patients had congenital elbow extension contractures: one unilateral and one bilateral. The triceps was lengthened in all three elbows and the elbow flexion restored as the first step to proceeding with ulnarization. In the one bilateral patient (Cases 11 and 12), both elbows were released with ulnarization simultaneously. In the unilateral patient (Case 17), this elbow procedure was performed 3 months after ulnarization at the same time as the wire removal and pollicization.

The HFA for all hands before surgery was a mean of 76.2° (range 12.4°–96.2°). The immediate postoperative HFA for this group measured a mean of 7.7° (range 0.2°–12.8°). The mean HFA correction was 68.5° (range 12.2°–88.7°). The mean ulnar bow preoperatively measured 17.2° (range 0.5°–64.3°). The mean ulnar bow postoperatively measured 4.8° (range 0.3°–25.7°).

The mean HFA for all 14 hands with longer follow-up before surgery was 82.4° (range 67.4°–96.8°) (Table 2). The immediate postoperative HFA for this group measured a mean of 8.9° (range 4.2°–12.7°) (Table 2). The late follow-up postoperative HFA for this group measured a mean of 11.4° (range 7.32°–14.4°). The mean HFA correction was 73.5° (range 58.2°–88.7°) (Table 2). Mean follow-up passive total wrist flexion–extension range of motion at the latest follow-up measured 76.6° (44°–88°) (Table 2). Recurrent radial deviation deformity was defined as an HFA angle increase of 15° or more. There were no recurrent radial deviation deformities (Table 2).

Mean ulnar length preoperatively measured a mean of 7.3 cm (range 5.7–9.3 cm). Mean ulnar length immediately after surgery measured a mean of 6.0 cm (range 4.1–7.6). Mean shortening during surgery was 1.4 cm (range 0.8–2.3) based on the difference between the pre- and postoperative lengths. Mean ulnar length at latest follow-up measured 7.2 cm (range 5.4–9.5 cm) (Table 3). Mean growth was 1.3 cm per year (range 0.2–2 cm) (Table 3). A Spearman test showed a statistically significant correlation between preoperative HFA and ulnar shortening (*p* = 0.01, r = 0.51).

## 4. Discussion

Paley et al. reported on a group of 21 RCHs treated by ulnarization G1 between 2000 and 2006 [15,17]. Mean age at surgery was 6 years (range 1–14). The follow-up range was 15–91 months (mean: 6 years). At the latest follow-up, there were no recurrent radial deviation deformity and no distal ulnar physeal growth arrest. There was an improvement in passive wrist dorsiflexion from a mean of 15 to 36 degrees. The HFA changed from a mean of 53 degrees radial to 22 degrees ulnar. Growth of the ulna, measured as an increase in ulnar length, changed from 79 mm after surgery to 102 mm at the latest follow-up. There were two wound complications that required additional surgery. The parents were all satisfied with the final appearance and function of the hand.

Long-term follow-up of the ulnarization G1 patients revealed that up to 15% developed a dynamic ulnar deviation of the hand [17]. This improved with active motion of the hand. This is likely due to gravity pulling on the unsupported hand at rest. The other late observation was a bump on the side of the hand due to prominence of the ulnar head. Radiographs demonstrated overgrowth of the ulna relative to the carpus and hand. A bumpectomy was performed in some children at the end of growth.

**Figure 4:**
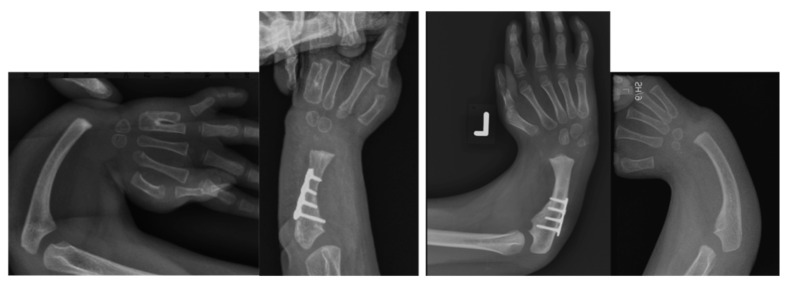
Case 9 left, 10 right. Bilateral RCH preop radiographs (left most, right most). Postop radiographs after ulnarization G3 of both hands (left middle, right middle). Note the hypertrophy of the head of the ulna after surgery in both hands.

**Figure 5:**
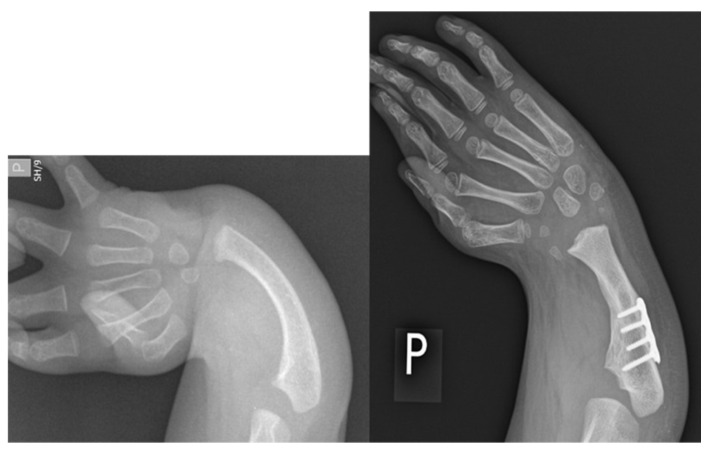
Case 6 preop radiographs (**left**); postop radiograph after ulnarization G3 (**right**). Note the hypertrophy of the head of the ulna after surgery.

In 2017, Paley described a second generation ulnarization procedure in which no external fixation was used, and the carpus was placed more on top of the ulnar head rather than on the side of the head of the ulna [17]. Subsequent follow-up of this group of patients, which is unpublished, showed that the head of the ulna continued to grow past the carpus in about 50%, although there were no recurrent deformities and few dynamic ulnar deviations. To address this, the senior author made a final modification of the procedure, placing the ulnar head into a more congruent pocket made up of the lunate and capitate on one side and the scaphoid on the other side, where the trapezoid or trapezium was on its end. This more contained, congruent and stable construct was created to prevent the ulna from growing past the carpus. The growth of the ulna past the carpus in Generations 1 and 2 is a testament to the unimpeded continued growth of the distal ulna with ulnarization. Ulnarization G3 maintains all the positive elements of the earlier generations of ulnarization with a more central location to the carpus. This may give it some of the advantages of centralization, namely, that the carpus sits directly on the end of the ulnar head for more direct loading and growth. Radiographically, ulnarization G3 is subjectively noted to show excellent hypertrophy of the end of the ulna, indicating the excellent loading of the ulna as one would see with centralization (Figure 4 and Figure 5).

To mitigate against recurrence with a more central location, the FCR, if present, is released, the ECU is shortened and the FCU transferred dorsally. The preliminary results of this study suggest that ulnarization G3 obtains excellent correction in all cases, as evidenced by the near complete acute correction of the hand–forearm angle and ulnar bow angle without the overcorrection seen in G1. In this short follow-up study, all cases showed less than 15° recurrence and the wrists were mobile and passively correctable. We are optimistic that G3 will follow the longer-term results of G1 and G2, in which there was no increase in recurrence rate. Longer-term follow-up is needed to confirm this. Even if mild recurrence occurs, as long as the wrist is passively correctible, it could be treated by repeat shortening of the ECU.

In 1998, Paley proposed distraction as a way to achieve the correction of a radial club hand [18]. The high recurrence rate with centralization has led some authors to try gradual distraction prior to centralization or radialization [19,20].

Murphy et al. [11] summarized the non-operative treatment vs. soft-tissue distraction with radialization or centralization in correction of wrist deformity in RCH. Patients treated with centralization had a greater improvement in the HFA of 71° compared with patients treated with radialization alone, which showed an improvement of 49°. Radialization maintained a better wrist ROM of 46° and ulnar length of 13.6 cm than centralization, with 25° and 11.5 cm, respectively. Both showed a greater HFA correction than in historical series of centralization without prior distraction.

Kanojia et al. [21] examined the outcomes of soft tissue distraction before centralization and transfer of flexor carpi radialis and flexor carpi ulnaris tendons in 18 hands. In 16 cases, the treatment was completed before 10 months of age. The results 31 months after surgery were good in seven, satisfactory in eight and unsatisfactory in one case.

Pfister et al. [22] retrospectively examined 31 hands in 28 patients who underwent progressive distraction and subsequent percutaneous pinning of the wrist with a corrective ulnar osteotomy. The HFA decreased from 64° to 12° after a mean follow-up of 7 years (range from 2 to 20). They reported 58 reoperations that were required in 31 wrists because of pin migration or breakage, and in addition 18 secondary osteotomies of the ulna were performed. They concluded that distraction provides satisfactory and stable realignment of the wrist to correct the deformity, but that this treatment has significant drawbacks regarding the high number of reoperations and the loss of wrist mobility.

Romana et al. [23] presented 13 patients treated sequentially, with distraction followed by centralization. HFA after centralization was reduced to a <12° mean. Ulnar osteotomy was required in eight cases (61%). One patient presented had a poor result due to insufficient coronal and sagittal correction.

Manske et al. [24] in 2014 carried out a study to evaluate the effect of soft-tissue distraction on recurrence of deformity after centralization for radial longitudinal deficiency. Thirteen upper limbs treated with centralization alone were compared with 13 treated with external fixator distraction followed by centralization with 2–10 years follow up. The authors observed centralization, with or without distraction, with an external fixator, corrected alignment of the wrist. Distraction facilitated centralization, but deformity recurrence was observed and associated with a worse radial deviation and volar subluxation position compared with wrists treated without predistraction.

Paley reported that distraction can lead to physiolysis of the distal ulna [18]. Furthermore, Paley observed that the length obtained in distraction was quickly lost in ulnarization G1, which led Paley to develop ulnarization G2, incorporating shortening [17]. Acute shortening removes all soft tissue tension and reduces the risk of recurrent deformity. For this reason, incorporating shortening of the ulna allows correction of very severe deformities and may also prevent recurrence by balancing the soft tissue tension [17]. Concern regarding shortening of an already short ulna is not merited. The length is already lost. The length of the forearm–hand unit is already short when one considers the axial length from the olecranon to the fingertips. After the correction, the axial length from the olecranon to the fingertips is longer despite the shortening. A better measure of length is the soft-tissue length of the ulnar vessels and nerve and of the median nerve. The length of these cannot change acutely. Viewed from the perspective of the nerves, the bone of the ulna is long. The shortening makes the lengths of the ulna and the nerves and vessels the same. This is the concept previously popularized by Paley, called relative length [25].

Damore et al. [26] presented 19 cases that had centralization, with an average follow-up of 6.5 years. Centralization corrected the preoperative HFA of 58° (range 15° to 95°) to 25° (range 5° to 60°). At the final follow-up examination, there was a loss of 38° (range 5° to 105°).

Lahiji et al. [27] presented 15 hands (13 patients) that underwent centralization with pollicization in a two-step approach, with soft tissue stretching and serial splinting before surgery. The mean HFA immediately after surgery was 13.8 ± 5°(range: 10°–23°) and increased on the final follow-up to 22.2 ± 13.5° (10°–60°). ROM in the sagittal and coronal plane of the operated hand was 83 ± 11% of the normal hand. Skin necrosis occurred in three patients.

Mazhar et al. [28] presented the results of 13 patients (16 hands) that underwent centralization. Patients were followed for 62.1 ± 39.9 months. The mean HFA correction was 29.4° ± 23.9°, and the mean HFA recurrence was 13.3° ± 13.7°. The mean ulnar bow corrected to 7.6° ± 12.5° immediately after surgery and a further 3.6° ± 7.3° at the last follow-up (overall 11.2° ± 17.6°).

Mittal et al. [29] did a randomized study comparing results of radialization. Radialization had a lower recurrence rate and lower growth arrest rate than centralization. Das et al. [30] compared ulnarization G2 and acute centralization. They found no difference between the functional outcomes of the two groups after one-year follow-up but did not comment on recurrent radial deviation or growth of the ulna between the two groups, except to say that two centralizations required distal ulnar resection. The distal ulnar resection follows the same relative length concept [25].

Bhat et al. [31] reported on radialization with a form of shortening called a metaphyseal cuff osteotomy with additional tendon balancing. They claimed that the distal shortening allowed them to correct the RCH deformity without damaging the epiphysis or the carpus This article supports Paley relative length concept of ulnar shortening for acute correction of RCH [17].

Vilkki et al. [32] described a microsurgical approach by using a vascularized second metatarsophalangeal (MTP) joint transfer for correction of RHD. Results of the mean follow-up of 11 years of 19 wrists in 18 patients showed an HFA of 28° with improved ulnar growth compared with centralization.

Yang et al. [33] showed 42 months of follow-up of four children, with mean age of 4.3 years, who underwent microsurgical reconstruction of the distal radius with vascularized proximal fibular transplantation. An average HFA correction of 28 degrees was obtained.

These last two methods require microvascular anastomosis and sacrifice of the second toe and metatarsal or fibular head. Such tissue transfer is not without significant morbidity. The results of all three generations of ulnarization offer a much lower morbidity procedure that can be performed by most hand surgeons without microvascular expertise and without sacrificing a toe or fibula.

Since ulnarization is a volarly performed radialization, the results of the radialization are more comparable to ulnarization than is centralization. The studies comparing radialization with centralization do support a lower incidence of recurrent radial deviation deformity and growth arrest with radialization. When considering the variable of acute shortening vs. distraction, the recurrence rate may be lower with acute shortening.

## 5. Conclusions

Ulnarization G1 and G2, and even the early results of G3 have a low risk of recurrence, growth arrest and wrist stiffness. In contrast, centralization and even distraction followed by centralization and radialization report high rates of these same complications. If these results are maintained with longer follow-up, consideration for replacing centralization with ulnarization may be in order.

## Figures and Tables

**Table 1:** Demographics and follow-up timeframe.

Case No.	Thumb	Syndrome	Sex	Involvement	Side	Age at Surgery (Months)	Follow-Up (Months)
1	Hypoplastic		Female	Unilateral	Right	37	19
2	Absent	VACTRL	Female	Unilateral	Left	28	19
3	Absent		Female	Unilateral	Right	29	19
4	Normal	TAR	Male	Bilateral	Left	32	19
5	Normal	TAR	Male	Bilateral	Right	32	19
6	Normal		Male	Unilateral	Right	18	19
7	Normal	TAR	Female	Bilateral	Left	19	15
8	Normal	TAR	Female	Bilateral	Right	19	15
9	Normal	TAR	Male	Bilateral	Left	25	15
10	Normal	TAR	Male	Bilateral	Right	25	15
11	Normal	TAR	Female	Bilateral	Left	17	15
12	Normal	TAR	Female	Bilateral	Right	17	15
13	Absent		Female	Unilateral	Left	26	12
14	Normal	TAR	Male	Unilateral	Right	21	12
15	Normal	TAR	Male	Bilateral	Left	23	8
16	Normal	TAR	Male	Bilateral	Right	23	8
17	Hypoplastic		Male	Unilateral	Right	25	8
18	Normal		Male	Unilateral	Left	18	7
19	Absent		Female	Unilateral	Right	19	7
20	Normal		Male	Unilateral	Right	21	3
21	Normal		Female	Unilateral	Left	18	3
22	Absent		Male	Unilateral	Right	52	3

VACTERL: vertebral–anal atresia–cardiac–tracheo–esophageal fistula–radius–limb; TAR: thrombocytopenia–absent radius.

**Table 2:** Data presents the HFA (hand–forearm angle) correction and postoperative combined range of motion.

Case No.	Total ROM (Flex. + Ext.)	HFA Pre-Op	HFA Post-Op	HFA Follow-Up	HFA Correction
1	83	76.2	12.8	14.4	63.5
2	44	88.4	8.9	12.4	79.5
3	76	85.1	10.9	11.3	74.1
4	69	95.8	9.4	12.4	86.4
5	56	82.2	6.3	9.1	75.9
6	98	76.3	10.7	13.4	65.7
7	64	96.2	7.4	9.2	88.7
8	78	95.2	9.7	12.2	85.5
9	86	67.4	9.1	10.2	58.3
10	90	81.0	7.5	13.2	73.5
11	78	68.1	9.9	12.4	58.2
12	92	74.7	8.1	9.4	66.6
13	84	92.4	9.2	12.9	83.2
14	75	74.3	4.2	7.3	70.1

**Table 3:** Data of the changes in the ulna regarding bowing and length.

Case No.	Ulnar Bow Pre-Op	Ulnar Bow Post-Op	Ulnar Length Pre-Op (cm)	Ulnar Length Post-Op (cm)	Ulnar Length at Follow-Up (cm)	Shortening (cm)	Growth (cm/year)
1	1.3	0.3	9.3	7.6	9.5	1.7	1.9
2	0.5	0.4	8.2	6.8	8.3	1.4	1.5
3	12.5	3.9	7.8	6.2	7.4	1.6	1.2
4	17.5	5.3	9.0	7.4	9.2	1.6	1.8
5	19.4	6.2	8.7	7.2	9.1	1.5	1.9
6	4.5	0.5	6.9	5,74	7.3	1.2	1.6
7	11.1	3.3	7.4	6.1	7.5	1.3	1.4
8	10.4	4.2	7.5	5.9	7.3	1.6	1.4
9	5.4	1.1	7.5	6.4	7.7	1.1	1.3
10	3.4	3.3	7.5	6.3	7.3	1.2	1.0
11	16.6	0.8	7.3	4.7	5.4	2.6	0.7
12	18.5	8.4	7.2	4.5	3.8	2.7	No Growth
13	5.5	6.2	7.9	6.2	6.9	1.7	0.7
14	7.3	8.2	8.1	7.1	8.1	1.1	1.1

## Data Availability

Not applicable.
